# Myocarditis and Sudden Cardiac Death in the Community: Clinical and Pathological Insights From a National Registry in the United Kingdom

**DOI:** 10.1161/CIRCEP.123.012129

**Published:** 2023-08-11

**Authors:** Raghav T. Bhatia, Gherardo Finocchiaro, Joseph Westaby, Nikhil Chatrath, Elijah R. Behr, Michael Papadakis, Sanjay Sharma, Mary N. Sheppard

**Affiliations:** Cardiovascular Clinical Academic Group and Cardiology Research Centre, St. George’s, University of London, St. George’s University Hospitals NHS Foundation Trust, United Kingdom (R.T.B., G.F., J.W., N.C., E.R.B., M.P., S.S., M.N.S.).; Hull University Teaching Hospitals NHS Trust, Kingston-upon-Hull, United Kingdom (R.T.B.).; Cardiovascular Pathology Department, St George’s, University of London, United Kingdom (J.W., M.N.S.).

**Keywords:** athletes, autopsy, myocarditis, sudden cardiac death, syncope

Myocarditis is an inflammatory cardiac disorder with variable clinical presentations and outcomes.^1,2^ Although some patients present with transient symptoms followed by rapid resolution, others may develop cardiogenic shock or fatal arrhythmias. A significant proportion of individuals die in the community and are diagnosed at autopsy^1–3^ raising questions about missed opportunities of a timely diagnosis for appropriate intervention to mitigate the risk of sudden cardiac death (SCD). We aimed to examine the presenting features and circumstances of death in a large cohort of decedents who experienced SCD in the community and were subsequently diagnosed with myocarditis at autopsy.

We reviewed 7702 consecutive cases of SCD referred to our specialist cardiac pathology center between 1994 and 2022. SCD was defined as death from a cardiovascular cause within 12-hours of apparent well-being. Clinical information was obtained from referring coroners, and included demographic characteristics of the deceased, medical history, family history, and circumstances of death. Cases underwent comprehensive autopsy evaluation of the heart, including histological analysis of a minimum of 10-tissue blocks, by expert cardiac pathologists (J.W. and M.N.S.).^4^ The diagnosis of myocarditis was based on the published histological criteria.^5^ Ethical approval was granted for this study (10/H0724/38). The data that support the findings of this study are available from the corresponding author on reasonable request. Results are expressed as mean±SD for continuous variables, or as number of cases and percentage for categorical variables. Comparison between variables was performed using 2 sample *t* test or Fisher exact test.

We only included deaths attributed to myocarditis in nonhospitalized individuals. Of the total cases of SCD, 82 (1.1%) were attributed to myocarditis (Figure). The majority of decedents were male (n=53 [65%]) with a mean age of death 32±15 years (range, 1–68 years). The mean heart weight was 378±128 g. Most individuals died at rest (n=73 [89%]), including during sleep (n=16 [19%]) while the remainder died during exertion (n=9 [11%]). Our cohort comprised 5 athletes (3 of whom died at rest and 2 during exertion). Only 1 individual was diagnosed with viral myocarditis, antemortem.

**Figure.**
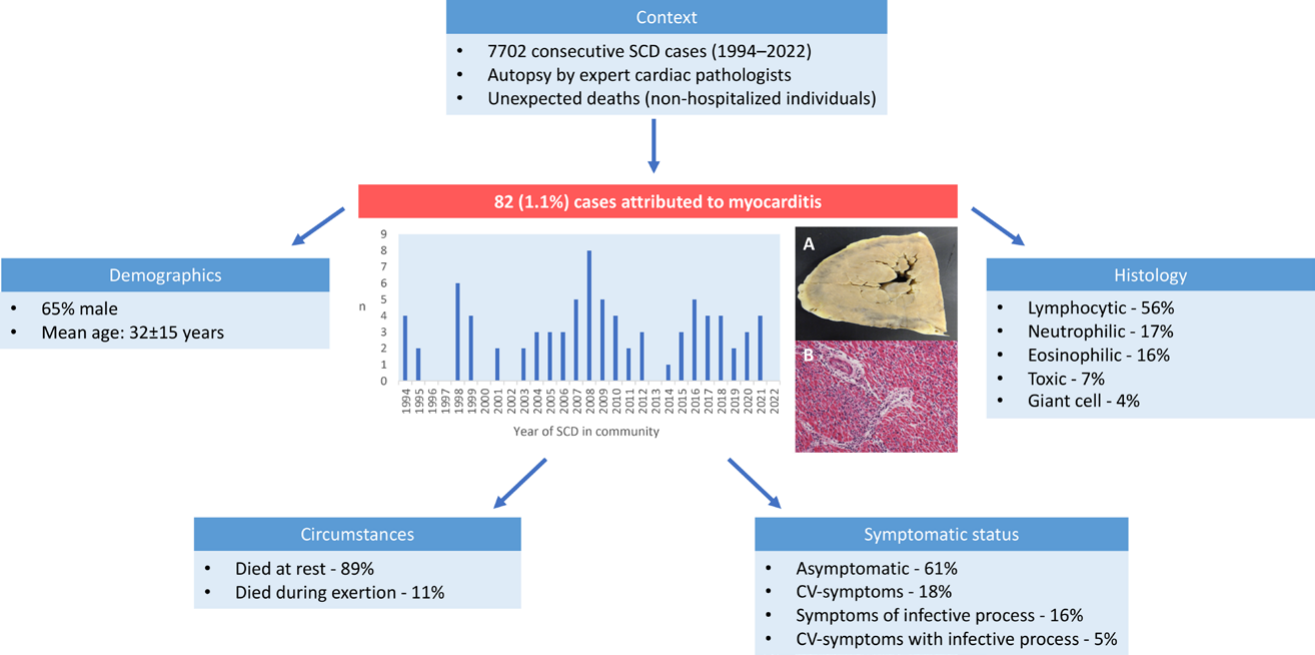
**Study overview.** Macroscopic (**A**) and microscopic appearances (**B**) of myocardium with lymphocytic myocarditis. The myocardium demonstrates mottling with dark discoloration (**A**) and evidence of a diffuse lymphoid infiltrate with corresponding myocyte necrosis (**B**). CV indicates cardiovascular; n, number of cases; and SCD, sudden cardiac death.

Most individuals (n=50 [61%]) were reportedly asymptomatic before SCD. Thirteen (16%) individuals reported symptoms consistent with an infective process in the 6 months before death. Cardiovascular symptoms were reported in 15 (18%) decedents, including dyspnea (n=6 [7%]), chest pain (n=5 [6%]), syncope (n=3 [4%]), and palpitation (n=1 [1%]). Four decedents (5%) reported coexisting cardiovascular symptoms in the context of ongoing infective symptoms. Lymphocytic myocarditis was the most common form, n=46 (56%); followed by, neutrophilic, n=14 (17%), eosinophilic, n=13 (16%), toxic, n=6 (7%), and giant cell myocarditis, n=3 (4%). There were no significant differences in the clinical or histopathologic findings between individuals who died at rest compared with individuals who died during activity.

Our observations show that myocarditis is a relatively rare cause of SCD in nonhospitalized individuals (1.1%) and the majority of decedents die at rest. Lymphocytic myocarditis was the most common form, which is consistent with data from studies based on the living individuals assessed with endomyocardial biopsies.^1,2,5^ Almost 1 in 5 individuals had prodromal cardiac symptoms; however, only 26% of these occurred in the context of an infective process. According to our results, only 5% of the decedents would have had a suspected diagnosis of myocarditis on the basis of cardiac symptoms in conjunction with an infective process, before SCD. Our findings highlight the challenges in identifying nonacute cases of an arrhythmogenic substrate in the community and emphasize the need for early diagnosis, which may be facilitated by a high index of suspicion of the disease and instigating rapid assessment and risk-stratification to reduce morbidity and mortality.

Our study has some limitations. Our referral center for cardiac pathology is more likely to receive hearts from subjects where the clinical history is suggestive of an inherited cardiac disease, or the autopsy findings are normal or equivocal; hence, our data may have underestimated the prevalence of SCD from myocarditis in nonhospitalized individuals. Nevertheless, we receive a high volume of unexpected SCD referrals (>400/y) and the large number of examinations performed in our unit suggests that the results are likely to represent the type and frequency of cardiac diseases implicated in SCD, especially in young individuals.

In conclusion, myocarditis accounts for 1% of all SCDs in nonhospitalized individuals. Cardiac symptoms preceding a fatality are noted in ≈1 in 5 individuals, although only 5% express cardiac symptoms in conjunction with infective symptoms.

## ARTICLE INFORMATION

### Acknowledgments

The authors thank Cardiac Risk in the Young (CRY), which fund and support the CRY Cardiovascular Pathology Unit.

### Sources of Funding

Drs Bhatia, Chatrath, and Finocchiaro are funded by a research grant from Cardiac Risk in the Young.

### Disclosures

None.
